# Myoepithelial carcinoma of the skin: a case report

**DOI:** 10.1093/jscr/rjaf151

**Published:** 2025-03-19

**Authors:** Sarra Ben Rejeb, Yasmine Chaabane, Amani Hachicha, Senda Turki

**Affiliations:** Pathology Department, Security Forces Hospital, Tahar Ben Achour Street, 1050, Marsa, Tunisia; Pathology Department, Security Forces Hospital, Tahar Ben Achour Street, 1050, Marsa, Tunisia; Otolaryngology Department, Security Forces Hospital, Tahar Ben Achour Street, 1050, Marsa, Tunisia; Otolaryngology Department, Security Forces Hospital, Tahar Ben Achour Street, 1050, Marsa, Tunisia

**Keywords:** myoepithelial carcinoma, cutaneous neoplasm, immunohistochemistry, differential diagnosis, surgical excision

## Abstract

Cutaneous myoepithelial carcinoma (MEC) is extremely rare and its diagnosis is challenging due to its broad morphological spectrum. We reported the case of a 50-year-old woman with a 7 mm cutaneous nodule on the left nasal wing, initially misdiagnosed as a fibrous papule. The lesion recurred 6 months after excision. Histopathological analysis revealed a dermal neoplasm with epithelioid cells exhibiting moderate atypia and mitotic activity. Immunohistochemical analysis confirmed a myoepithelial phenotype with positive staining for CK7 and S-100, while excluding adnexal, vascular, muscular, histiocytic, plasmacytic, and melanocytic origins. A diagnosis of primary cutaneous MEC was established. The patient underwent complete surgical excision with clear margins, and no recurrence or metastasis was observed after 6 years of follow-up. This case highlights the diagnostic challenges associated with primary cutaneous MEC and underscores the importance of complete surgical excision. Increased awareness and further case reporting are necessary to refine diagnostic and therapeutic approaches for this rare malignancy.

## Introduction

Myoepithelial carcinoma (MEC) is a rare and highly aggressive malignant tumor originating from myoepithelial cells [1]. It most commonly occurs in the salivary glands, accounting for ⁓4% of salivary gland neoplasms [1]. Cutaneous MEC is even rarer, with only 14 cases reported in the literature [2]. It must be distinguished from soft tissue myoepithelial carcinomas with subcutaneous involvement. Diagnosing cutaneous MEC is challenging due to its broad morphological spectrum, which may mimic other myoepithelial or cutaneous tumors [1]. We herein presented a rare case of primary cutaneous myoepithelial carcinoma and discussed the diagnostic challenges.

## Case presentation

A 50-year-old woman with no significant medical history presented with a well-circumscribed, flesh-colored, shiny cutaneous nodule measuring 7 mm on the left nasal wing. Physical examination was unremarkable, especially no palpable masses or lymph nodes in the head or neck region. Initially diagnosed as a fibrous papule of the nose, the tumor recurred 6 months later.

**Figure 1:**
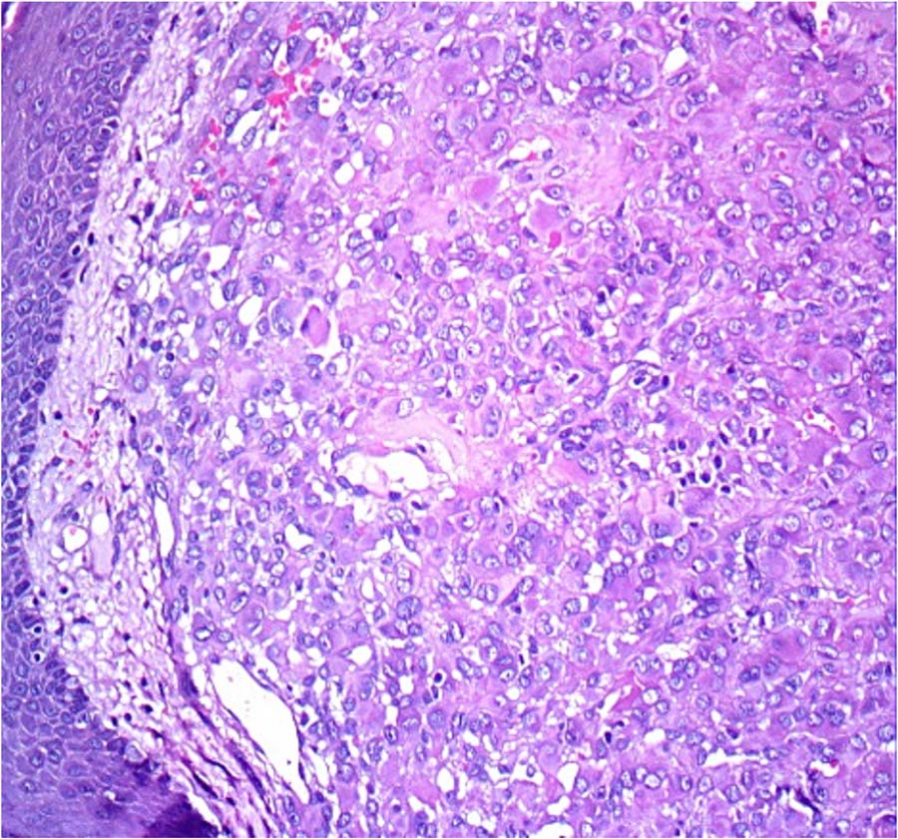
HE ×20: A diffuse poorly differentiated neoplasm composed of pleomorphic epithelioid cells.

**Figure 2:**
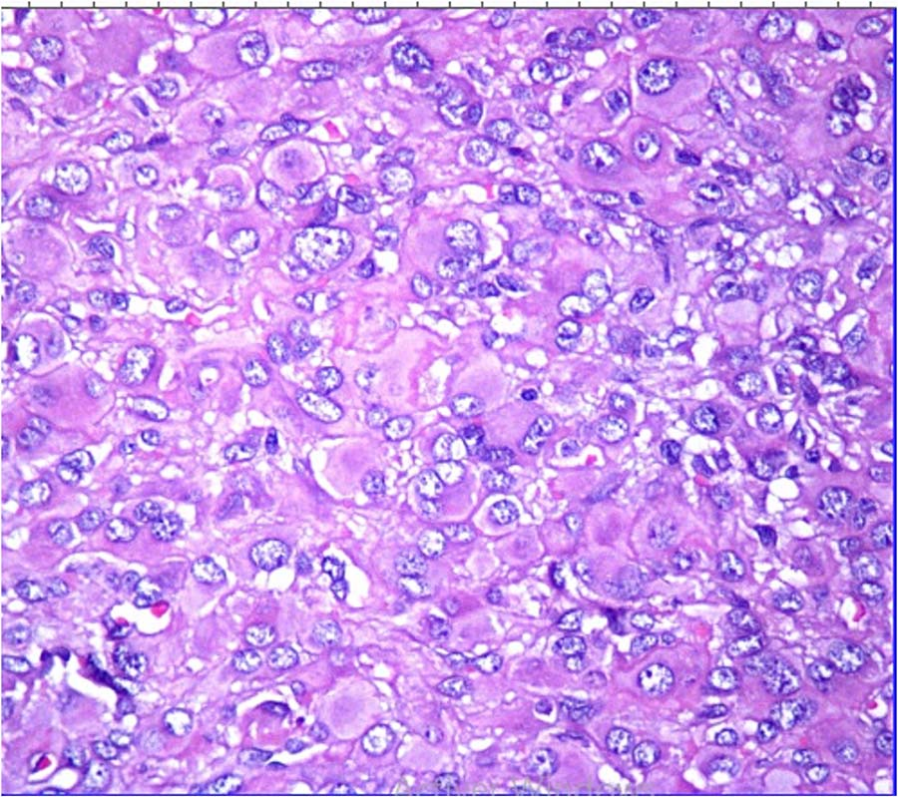
HE ×40: The tumor cells are epithelioid with atypical nuclei.

Histopathological analysis revealed a poorly differentiated dermal neoplasm arranged in a solid growth pattern. The tumor cells were epithelioid, with abundant eosinophilic cytoplasm and centrally or eccentrically placed nuclei exhibiting moderate atypia and prominent nucleoli (Figs 1 and 2). The mitotic rate was 4 per 10 high-power fields (HPF). The stroma was fibrous, and the surgical margins were involved. Immunohistochemical analysis showed tumor cells positive for vimentin, CK7, and S100 protein, with a MIB-1 proliferation index of 5%. Negative staining for cytokeratin, androgen receptors, PHLDA1, and p63 excluded adnexal tumors. Additionally, vascular markers (CD34, CD31, ERG), muscular markers (desmin, caldesmon, actin, myogenin), histiocytic markers (CD68, CD163, MMP11), plasmacytic markers (CD138, MUM1), and melanocytic markers (Melan-A, HMB45) were negative.

Based on the morphological and immunohistochemical findings, a diagnosis of myoepithelial carcinoma was established. Brain MRI ruled out possible tumor extension, lymph nodes or additional lesions elsewhere. A final diagnosis of primary cutaneous myoepithelial carcinoma was confirmed. Complete surgical re-excision with clear margins was performed. No recurrence or metastasis was observed after 6 years of follow-up.

## Discussion

Myoepithelial cells are specialized contractile cells with both epithelial and mesenchymal characteristics [1]. Myoepithelial tumors primarily occur in the salivary glands, and cutaneous presentations are rare, particularly the malignant variant MEC. To date, only 15 cases of primary cutaneous myoepithelial carcinoma, including the present case, have been reported [2]. Given its rarity, clinical presentation is not well-characterized, and MEC may be mistaken for benign lesions such as dermatofibromas or seborrheic keratoses.

Most reported cases, including ours, involved female patients, suggesting a slight female predominance [2–10]. The reported age range is 2.5–85 years, with a median age of 50 years. Lesions typically present as slow-growing, nodular skin masses that are often asymptomatic.

Histopathologically, MEC exhibits significant morphological heterogeneity. Tumor cells may appear spindle-shaped or epithelioid, with eosinophilic or clear cytoplasm and varying degrees of nuclear atypia. The stroma can be loose, myxoid, or collagenous, with occasional central necrosis. Cytologic atypia, high mitotic activity, and tissue infiltration are indicators of malignancy [1, 2].

Kutzner et al. proposed histopathological criteria favoring a benign myoepithelial tumor: a well-circumscribed dermal nodule without epidermal connection, a solid appearance with cellular aggregation, monomorphic neoplastic cells with ovoid nuclei and pale eosinophilic cytoplasm, and hyalinized or myxoid stroma [5]. On the other hand, infiltrative borders, atypical nuclei, mitosis and necrosis are more common in malignant counterparts. Immunohistochemistry is crucial for confirming the myoepithelial origin, as neoplastic cells typically express both myogenic (desmin, myogenin, actin) and epithelial markers (EMA, CK), with consistent S-100 immunoreactivity [1, 2].

In this described case, the tumor exhibited pleomorphic epithelioid cells in a diffuse and poorly differentiated pattern. Immunohistochemical analysis excluded adnexal, vascular, muscular, histiocytic, plasmacytic, and melanocytic origins. The combined positivity for CK and S-100 supported a biphasic epithelial and myoepithelial differentiation. Despite a low proliferative index, the marked cytological atypia and tumor recurrence were indicative of malignancy.

This case report increases awareness among pathologists to always consider myoepithelial carcinoma in the differential diagnosis of cutaneous neoplasms composed of both epithelioid and spindle shaped cells.

Given the nonspecific histological findings, the diagnosis of MEC should be primarily based on immunohistochemical profiling. It is essential to distinguish primary cutaneous MEC from soft tissue variants, which are typically deep-seated and require more extensive surgical resection [1, 2]. Although the anatomical distribution and immunohistochemical profiles are similar, cutaneous MEC appears to have a lower recurrence rate than its soft tissue counterpart [2, 7].

Complete surgical excision with clear margins remains the gold-standard treatment for MEC [2–10]. In the present case, the first resection was incomplete with positive margins which led to tumor recurrence. Postoperative radiotherapy is recommended for cases with close margins or lymph node metastases. However, cases with distant metastases often follow a more aggressive clinical course, with a generally poor prognosis [2]. Nevertheless, even in cases of inappropriate resection or local recurrence, if complete resection is ultimately achieved prior to clinical metastasis, the prognosis is good and survival is prolonged [2–10]. Therefore, obtaining complete resection appears to be the most important treatment strategy for primary cutaneous myoepithelial carcinoma.

## Conclusion

Primary MEC is a very rare and challenging diagnosis due to its non-specific histology and overlapping features with other cutaneous and myoepithelial neoplasms. This case highlights the importance of thorough histopathological examination and immunohistochemical profiling in differentiating MEC from adnexal, vascular, muscular, histiocytic, and melanocytic tumors. Given its potential for local recurrence, complete surgical excision with negative margins is the treatment of choice. Although cutaneous MEC appears to have a lower recurrence rate than its soft tissue counterpart, long-term follow-up remains essential to monitor for potential recurrence or metastasis. Increased awareness and reporting of such cases will help refine diagnostic criteria and therapeutic strategies for this rare malignancy.
